# Uncovering the Properties of Energy-Weighted Conformation Space Networks with a Hydrophobic-Hydrophilic Model

**DOI:** 10.3390/ijms10041808

**Published:** 2009-04-21

**Authors:** Zaizhi Lai, Jiguo Su, Weizu Chen, Cunxin Wang

**Affiliations:** 1 College of Life Science and Bioengineering, Beijing University of Technology, Beijing, 100124, P.R. China; E-Mails: laizaizhi@emails.bjut.edu.cn (Z.L.); jiguosu@emails.bjut.edu.cn (J.S.); wzchen@bjut.edu.cn (W.C.); 2 College of Science, Yanshan University, Qinhuangdao, 066004, P.R. China

**Keywords:** Protein Folding, Conformation Space, Complex Network

## Abstract

The conformation spaces generated by short hydrophobic-hydrophilic (HP) lattice chains are mapped to conformation space networks (CSNs). The vertices (nodes) of the network are the conformations and the links are the transitions between them. It has been found that these networks have “small-world” properties without considering the interaction energy of the monomers in the chain, i. e. the hydrophobic or hydrophilic amino acids inside the chain. When the weight based on the interaction energy of the monomers in the chain is added to the CSNs, it is found that the weighted networks show the “scale-free” characteristic. In addition, it reveals that there is a connection between the scale-free property of the weighted CSN and the folding dynamics of the chain by investigating the relationship between the scale-free structure of the weighted CSN and the noted parameter *Z* score. Moreover, the modular (community) structure of weighted CSNs is also studied. These results are helpful to understand the topological properties of the CSN and the underlying free-energy landscapes.

## 1. Introduction

The complex network model [1–4] has been largely used to study complex systems, such as atomic clusters [5], polymers and proteins [6]. The structure and dynamics of these systems are commonly complex [7] and they always involve a large number of degrees of freedom. For example, protein folding is a complex process that can be described well by the theory of the free-energy landscape [8,9], which has also been successfully applied to the study of a broad range of other systems [10,11]. However, due to the complexity of the systems or the processes and the large number of degrees of the freedom involved, a detailed, impartial description of the free-energy landscape underlying the thermodynamics and kinetics cannot easily be extracted. To tackle the problem, new methods based on complex networks have recently been investigated. Doye and Massen have applied a complex network method to research potential energy minima of the small Lennard-Jones clusters [5,12]. In another work, the concept of disconnectivity graphs has been applied to analyze the free energy of a peptide [13,14]. Besides, the structure of the conformation space (the collection of all possible spatial conformations) based on a lattice model was also studied [15]. In addition, the free-energy landscape of a three-stranded *β*-sheet (beta3s) and alanine dipeptide have been represented as a conformation space network (CSN) [6,16], which is constructed based on the ensemble of microstates (conformations) and their dynamic connectivity. It has been shown [5,6,12,16] that the CSN of a complex system is an useful tool to study the topology of the conformational space and the dynamical connectivity of the conformations.

In reference [15], the conformation space of a short two-dimensional (2D) lattice polymer chain was mapped to a network where a link between two nodes indicates the interconversion in a single Monte Carlo move of the chain. Scala *et al*. found that the network was a small-world network [2] without a scale-free structure [3]. The study of their work relied on two important simplifications. The first was to use a lattice model, and the second was to neglect the interaction energy of the monomers in the chain [15]. Neglecting such interaction means that every conformation has the same weight and the topology of the network is determined by the connectivity of the conformation space. In this paper, the weight based on the interaction energy of the monomers in the chain is added to the CSNs and the weighted CSNs are constructed. Through analyzing the weighted networks, it is found that the CSNs show the “scale-free” property, that is, the weight distributions of the CSNs have the property of power-law tail (~ *w*^−^*^γ^*), where *w* is the energy weight of the vertex, and *γ* is the scaling exponent. This result indicates that the interaction energy of the monomers in the chain plays an important role in shaping the topology of CSN, thus perhaps providing a possible explanation [17] for the origin of the scale-free property of the CSN.

Furthermore, the power-law property of the CSNs may have an important relationship with the dynamics of some complex processes such as protein folding. As we know, among the all the possible linear amino-acid sequences, only very few are “protein-like” [18]. Such sequences must not only be thermodynamically stable, but also have a relatively fast folding time and should be stable against mutations [19]. Therefore, folding rate is one of the key factors that determine the sequences to fold to the ground state successfully [20]. Under the pressure of evolution, a protein-like sequence should have a relatively fast folding time [21]. And the topology of CSN may influent the folding rate. In other words, the sequences with different folding rate may show different properties of the CSN. To probe the connections, we investigated the relationship between the scaling exponent *γ* and the *Z* score [19,22–24], which was often used to measure how much a sequence is “protein-like” [24]. It is found that they have distinct correlation. Considering that the *Z* score is a good touchstone for the thermodynamic stability and kinetic of the sequences [19,24], the result implies that the topology of the weighted CSN is of deep connection with the folding dynamics of the sequences.

Finally, the modular (community) structure [25] of the weighted CSNs is also investigated. The concept of community means the appearance of the densely connected subsets of vertices, with only scanty connections between subsets in a complex network [25]. Such structure of complex network has been reported not only in social networks [25], but also in biochemical networks [6,13,26,27], food webs [28], and the internet [29]. To study this interesting property, several methods have also been developed recently to find the meaningful division of a network [30,31,32]. Simultaneously, it is widely accepted that the modular structure of complex networks plays an important role in their functionalities [33]. Therefore, the modular analysis of the weighted CSNs will be helpful for us to uncover the main features of the conformation space and the underlying energy landscape at a more coarse-grained level, thus reducing the complexity of the problem [34]. Especially, it will help us to discriminate between ‘easy folder’ proteins from those having a large number of folding traps.

## 2. Model and Method

### 2.1. Construction of the weighted conformation space network

Here the weighted CSN was studied with the 2D hydrophobic-hydrophilic (HP) square lattice model proposed by Dill [35]. The main point of that model is that the interactions between hydrophobic amino acids are the major driving force in protein folding. There are many advantages to using the HP model. First of all, complete enumeration of the sequence space and the conformation space is possible for sequences of given length. In our work, all sequences have a length of 13. Moreover, the definition of the interaction potential of the conformation is simple. In the present study, the interaction energy *E* of a conformation was defined as the number of topological contacts between adjacent hydrophobic amino acids that were not neighbors in the sequence, denoted by *E_HH_* *=* −*1*, *E_PP_* *= E_HP_* *= 0*. It is common practice in the HP model to simply regard *E* as an “energy”, and neglect the fact that it is more correctly a free energy [35,36].

**Figure 1.**
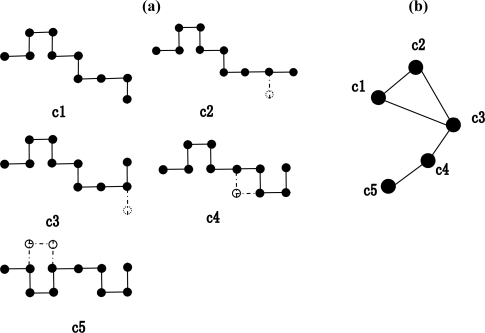
(a) The example of Monte Carlo move. The five different conformations of a 10 monomers lattice chain are labeled **c1** to **c5**. The dark circles represent the moving monomers. The dashed lines and circles with no shade represent the position of the monomer moved from the previous conformation. (b) The construction of the unweighted network.

First, the conformation space was mapped onto an unweighted network. All allowed conformations of a chain, which has unique ground-state conformation, were enumerated. Conformation of the chain was evolved by the conventional Monte Carlo elementary moves [37]: the end rotation, the corner flips and the crankshaft move. As shown in Figure 1(a), the end monomer can be rotated either 90° (conformation **c1** switches to conformation **c2**) or 180° (conformation **c1** switches to conformation **c3**); conformation **c3** can switch to **c4** by single corner flips; and conformation **c4** translates to conformation **c5** by the “crankshaft” move, which can move the two monomers at the same time. All moves must obey the excluded volume criteria: no lattice sites can be doubly occupied. Then, the conformation space can be mapped to an unweighted network. Each conformation of the chain is identified as a vertex of the network. A link is created between two vertices if the two corresponding conformations translate through a single elementary Monte Carlo move. As seen in Figure 1(b), since the conformation **c1** can be switched to the conformation **c2** by a single Monte Carlo move, so there is a link between them. However, the conformation **c2** can not be switched to the conformation **c4** by a single Monte Carlo move, so that there is no link between them.

Second, the energy weight was added to the network. According to the interaction energy of the conformation, the Boltzmann factor of each conformation *exp*[−*E* / *k*_B_*T*] can be calculated, where *E* and *k*_B_ are the energy of the conformation and the Boltzmann constant, respectively, and *T* is the absolute temperature. We set *k*_B_ = 1 and *T* = 1 in this work. The energy weight *W_i_* of the vertex *i* in the networks, represents the importance of *i*-th conformation in the conformation space, which can be defined as following: assuming that the vertex *i* connects with vertices *j*, *k*, *h*, the sum of the Boltzmann factors of the vertices *j*, *k*, *h* is treated as the weight of the vertex *i.* Obviously, the numerical value of the weight of the vertex *i* is a real number. Therefore, a vertex with the weight *w* means the numerical value of the vertex’s energy weight falls into the half open interval [*w*-1, *w*).

Finally, we chose HP sequences to construct the weighted CSNs. As to the HP model, the relationship of the conformation–sequence has been studied thoroughly [38], and the report that the features of protein folding are weakly dependent on the chain length [39,40] and the properties of protein folding tested by using 2D lattice model and 3D lattice model are similar [36,37] may provide confidence of the validity of using short chain models to study the folding problem. Here we started from a set of 2^13^ HP sequences. Through searching the complete conformations of each sequence, a set of 309 sequences with unique lowest-energy state was found from the 2^13^ HP sequences. A natural protein sequence has a unique global minimum of free energy which is well separated in energy from other misfolded states. In a lattice HP model, the protein-like folds are associated with sequences that have a minimal number of lowest-energy states [41,42]. And the sequences with only one global ground state candidate are good sequences [43]. The native structures of these sequences have well stability and designability [19,43]. We randomly chose three short sequences from these 309 sequences in order to exhibit the scale-free property of the energy-weighted CSNs. The first sequence was (HHPPPHPHPPHPH), and the other two sequences were (HHHHHPHHPPPHP) and (HHHPPPHHPHHHP).

### 2.2. Folding dynamics and the power-law property of the weighted CSNs

To uncover the relationship between the folding dynamics and the power-law property of the weighted CSN, the set of 309 sequences, as mentioned above, with unique lowest-energy state was applied. Then according to the method of the construction of the complex network above, we constructed 309 weighted complex networks. It is found out that all of these weighted networks show power-law tail properties, thus we can obtain all the scaling exponent *γ* s of the weighted CSNs.

Additionally, a known parameter, Z score (*Z* = Δ/Γ), was introduced in this article. In the *Z* score expression, Δ denotes the average energy difference between the ground state and all the other states and Γ is the standard deviation of the energy spectrum, which can be expressed as follows [19]:

(1) $$\Delta =\frac{1}{N_{C}}\sum_{\alpha >0}(E_{\alpha }-E_{0}),$$

(2) $$\Gamma^{2}=\frac{1}{N_{C}}\sum_{\alpha >0}E_{\alpha }^{2}-{\left(\frac{1}{N_{C}}\sum_{\alpha >0}E_{\alpha }\right)}^{2},$$

where *E_a_* (*α* > 0) represents the energies of the excited conformation, and *E_0_* is the lowest state energy, and *N_C_* is the number of excited compact conformations. According to the Equations (1) and (2), we can get all values of the Δ/Γ of the conformation spaces generated by the sequences that have unique ground-state. Then the relationship between the exponent scale *γ* and *Z* score can be studied.

### 2.3. Modularity-detection algorithm

In this article, a multistep greedy algorithm (MSG) in combination with a local refinement procedure named “vertex mover” (VM) [31,32] were applied to detect the module structure of the weighted CSNs. The MSG-VM algorithm is an agglomerative hierarchical clustering method and is based on the optimization of the modularity function that is defined as:

(3) $$Q=\sum_{i=1}^{N_{C}}\left[\frac{I(i)}{L}-{\left(\frac{d_{i}}{2L}\right)}^{2}\right],$$

with *I*(*i*) the weights of all edges linking pairs of vertices in community *i*, *d_i_* the sum over all degrees of vertices in module *i*, *L* the total weight of all edges, and *N_C_* the number of community. At the first step of the algorithm, each vertex in the network is considered as a community. Then the process of the algorithm involves finding the changes in *Q* that would result from the amalgamation of communities, selecting the largest of them. The MSG algorithm is to allow the merging of *l* (*l* > 1) pairs of communities at each iteration step, developing the algorithm that can only merge a pair of communities at each iteration step [30].

The algorithm works as follows: i. Start with the modularity change matrix Δ*Q*, whose initial value is $Q_{0}\;=\;-\sum_{i=0}^{N}\frac{d_{i}^{2}}{4L^{2}}$ (the means of *d_i_* and *L* are the same in the expression (3). N is the number of vertices.); ii. Change the initial values of Δ*Q* according to $\Delta Q_{ij}\;=\;\frac{I}{L}\;-\;\frac{d_{i}d_{j}}{2L^{2}}$ with I the weight of the edges connection the vertices *i* and *j*, *d_x_* the degree of vertex *x* = *i*, *j*, and L the total edge weight; iii. Select the elements with among highest *l* values, then join the corresponding communities, and update the matrix Δ*Q_ij_*; iv. Repeat the third step until getting the best values of modularity.

In the MSG-VM algorithms, the value of *l* and the weight of each edge of the network should be set. Here, an empirical formula was applied [32] for the choice of the step width *l*, which can be expressed as follows:

(4) $$l=\alpha \sqrt{L},$$

where *L* is the total weight of the edges and *α* = 0.25 [32]. On the other hand, we defined the weight of the edge in the network as follows: assuming there is an edge *e* between two nodes *n*_1_ and *n*_2_, the product of the Boltzmann factors of the two nodes *n*_1_ and *n*_2_ is defined as the weight of edge *e*, which is given by:

(5) $$W_{e}=\text{exp}[-(E_{n_{1}}+E_{n_{2}})/k_{B}T],$$

where *W_e_* is the weight of the edge *e; E*_*n*_1__ and *E*_*n*_2__ are the energies of the conformations which correspond to nodes n_1_ and n_2,_ respectively; *k*_B_ is the Boltzmann constant; *T* is the absolute temperature. We set *k*_B_ = 1 and *T* = 1 in this work.

## 3. Results and Discussion

### 3.1. The power-law property of the weighted CSN

For a complex network, the widely studied characteristic is the degree. Degree of a vertex is the total number of its connections. This quantity is also called “connectivity”. The degree of a vertex is a local quantity, and the total distribution of vertex degrees often determines some important global characteristics of the network. As to different kinds of networks, the degree distributions follow different forms. The degree distribution of a scale-free network is of a fat-tailed form [44,45]. A good approximation of the degree distribution of a random network is a binomial distribution, which can be replaced by Poission distribution for large number of vertices [4]. In this paper, each vertex in the network was endowed with an energy weight, so the weight is a basic quantity of a vertex, and the weight distribution is an important quality of the weighted network.

**Figure 2.**
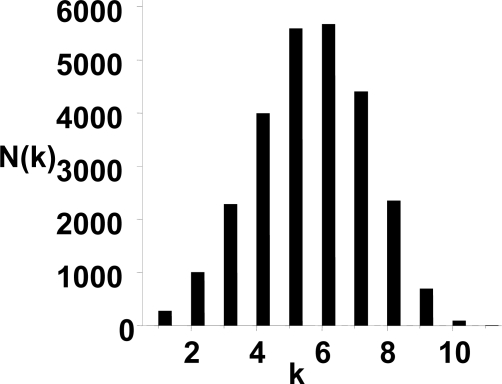
The connectivity distribution of the unweighted conformation space network. In this figure, **k** is the number of the connectivity and **N(k)** is the number of the vertices for the connectivity of **k.**

**Figure 3.**
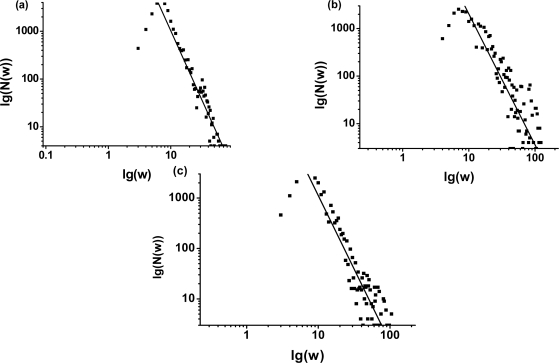
Topological properties of weighted conformation space networks. Logarithmic coordinate is used. **w** is the energy weight of a vertex, **N(w)** is the number of the vertices whose numerical value of the energy weight fall into the half open interval [**w-1**,**w**). The figures (a), (b) and (c) show the weight distributions of conformation space networks for the 3 sequences: (a) (HHPPPHPHPPHPH); (b) (HHHHHPHHPPPHP); (c) (HHHPPPHHPHHHP). The straight lines correspond to a power-law fit ~ *w* ^−^*^γ^* on the tail of the distribution of the weighted CSNs, where *w* is the energy weight of the vertex, and *γ* is the scaling exponent.

Figure 2 shows the connectivity distribution of the unweighted CSN. Obviously, the connectivity distribution agrees well with the Poission distribution, thus the topology of the unweighted network is relatively homogeneous, with most of nodes having approximately the same number of the edges. The result is consistent with the previous work [15]. In Figures 3(a)–(c), it is found that the weight distributions of the CSNs show a well defined fat-tail property. The continuous lines correspond the straight-line fit ~ *w* ^−^*^γ^* on the tail of the weight distribution, where *w* is the energy weight of the vertex, and *γ* is the scaling exponent. Furthermore, we also investigated the small-world property of the weighted CSNs.

Using the edge weight defined as (5), we firstly calculated the average shortest distance *l_w_* of the weighted CSN and compared it with that of random network *l_random_* ~ ln(*n*)/ln(〈*k*〉) [4], where 〈*k*〉 is average degree of the CSN, and *n* is the number of the nodes in the CSN. Then we randomly chose 10 weighted CSNs from the whole 309 weighted CSNs as the case study. The results shows that the *l_w_* of these weighted CSNs are all around 60 (data not shown), which is similar with the previous work [15]. The results also indicate that the *l_w_* of the weighted CSNs is larger than *l_random_* and is of the same magnitude of the *l_random_*. In our model, we observed *l_random_* ≈ 10. Secondly, we estimated the clustering coefficient *C_w_* of the weighted CSNs, obtaining that $\frac{C_{w}}{C_{\text{random}}}$: 10^2^, where *C_random_* ≈ 〈*k*〉/*n* is the clustering coefficient of random network. This result indicates that *C_w_* is much larger than *C_random_*. In summary, the above results suggest that the weighted CSNs have scale-free and small-world properties [2].

The result indicates that the energy weight is a key factor for the appearance of the power-law property of the CSN. To understand the meaning of the energy weight, we should consider the connectivity of the conformations and more details about the lattice model. It is well known that the lattice model is a simplified model of a polymer. In the lattice model, polymers including proteins can be treated as linear strings of beads, and the details of the structures are not accurately represented. This simplified approximation is based on the following hypothesis: when the real chain conformations are under good solvent conditions or in “theta”[46] (where conformations are highly expanded), the structural details such as side chain are subsumed under the property of chain stiffness [47], in other words, some of the degrees of freedom of the structures are “frozen”, but an actual polymer chain is flexible in the three-dimension Euclidean space. For a given stable conformation, due to the influence of the structure details such as the side chain, there are small conformational fluctuations around the stable conformation [47–51]. As to the lattice model, it means that for a given lattice conformation, there are many tiny difference conformations fluctuating around this conformation. In other words, there is an ensemble of subconformations belonging to the given lattice conformation. Further, the Boltzmann factor of a conformation incarnates the relative quantity of the subconformations of the ensemble [16,52]. On the other hand, the properties of some dynamical processes, such as protein folding, are determined not by the spectrum but also by the connectivity of the conformations [53]. If the energy barrier between the two conformations is too high, then we can say that the two conformations are not “dynamically connected” [8]. If a conformation is not dynamically connected to other low energy structures, then it would be kinetically inaccessible in spite of its low energy [8,54]. Therefore, assuming that the vertex *i* connects with vertices *j*, *k*, *h*, the weight of vertex *i*, as mentioned above, may reflects the number of all possible potential dynamically connected subconformations connecting to the conformation that corresponds to the vertex *i*. In other words, the weight of a node in the CSN represents the number of possible potential links connecting the node, implying the importance of the node in the CSN.

When the interaction energy of the monomers in the chain is not taken into account, all conformations are of the same free energy, i.e. unit free energy. It means that each conformation has the same weight in the conformation space. Therefore, the topology of the CSN is simply determined by the connectivity of the conformations. In this case, the degree distribution of the network is binomial distribution and significantly deviates from the power-law form, as shown in Figure 2. In terms of the free energy landscape, the free energy surface is flat [5] and the power-low property disappears.

The power-law distribution indicates that there are a few “hubs” which have large connectivity and a mass of vertices with a relatively small number of links in the network [3]. In the weighted network of conformation space, the power-law property indicates that a few conformations play the role as “hubs” in the dynamic process of complex systems, such as protein folding. The large weight of a conformation shows that this conformation has relatively more connections and the energies of its connective conformations are low. According to the definition of the energy weight, the large weight of a conformation implies that there are abundant possible routes to access it.

### 3.2. The scaling exponent γ s versus the ratio Δ/Γ

**Figure 4.**
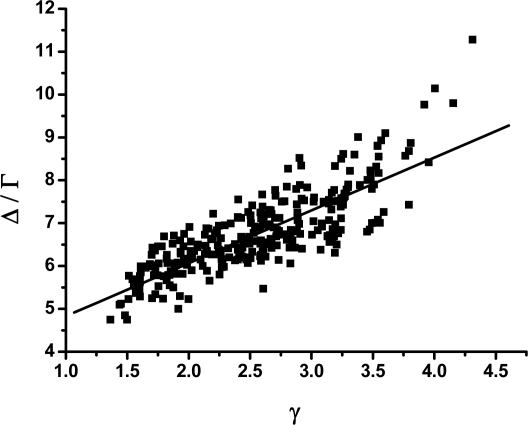
The scaling exponent *γ* s of the weighted CSNs *versus* the ratio Δ/Γ. The continuous line represents the straight-line fitting of the data (with a correlation coefficient of 0.81).

Figure 4 shows the relation between *γ* and Δ/Γ, in which there is high correlation between *γ* and Δ/Γ. It has been reported that *Z* score correlates significantly with the folding rates [55,56] and the sequences with a large ratio Δ/Γ fold fast [19,57,58]. Therefore, the large value of *γ* implies the fast folding.

This result can be understood by the free-energy landscape view. According to the mathematical knowledge of the power-law function [59], the larger scaling exponent indicates that the value of the function decays to zero more quickly for the relatively large independent variable. For example, for the power-law function *f* (*x*) = *x* ^−^*^β^* (*β* > 0), the larger value of *β* indicates that the value of *f* (*x*) inclines to zero more quickly when *x* → +∞. This property can be applied to analyze to the weight distribution of the weighted CSN. As expressed above, the weight distribution of the weighted CSN is a function, which gives the possibility that a randomly selected node has a definite weight. This function is of power-law property and *γ* is its scaling exponent. So, the larger scaling exponent *γ* indicates the less possibility finding the nodes with large weight. In other words, for the larger *γ*, there is relatively less number of nodes which have large weight. On the other hand, according to the physical meaning of the weight of the nodes, the large weight of a node means that this node has dense connections around it. Therefore, a lesser number of nodes with large weight in the network signify there are fewer crowded groups in the network. In terms of free-energy landscape, this scenario implies that the free-energy surface has a small number of deep valleys and high barriers between them [5,13]. So, the larger *γ* indicates that the free-energy landscape is smoother [8].

It has been shown [19,24] that the ratio Δ/Γ can be used to measure how much a sequence is “protein-like”, in other words, it is a good criteria for the thermodynamic stability or the stability of against mutation of the sequences. Simultaneously, the similar perspective has been reflected recently in the research of biomolecular binding [60] and cellular networks [61,62] in which the ratio of the energy gap versus roughness of the underlying energy landscape, which in essential is equivalent to the ratio Δ/Γ, has been recognized as an optimization criteria for the specificity of binding in binding energy landscape or the global thermodynamic stability (or robustness) of the network. The relationship between Δ/Γ and *γ* shown in Figure (4) suggests that the scaling exponent of the power-law of the networks should be considered carefully as a valuable topological parameter for the thermodynamic stability (or the robustness) of the CSN, which potentially may be good news for the design of artificial networks.

This result is also helpful for protein design, whose goal is to identify amino acid sequences that can fold well and lead to a given structure [63,64]. It has been shown [65,66] that for the *Z* score, which was always defined to have negative value in those papers, minimization is equivalent to maximizing the energy gap between mis-folded or unfolded conformations and the native state of the proteins, and such maximization results in stable and fast-folding proteins. Optimization of the *Z* score thus provides a quantitative method for the problem of protein design and has been widely applied [52,67,68]. Therefore, considering the result shown in Figure (4), the scaling exponent *γ* may provide an alternative optimization criterion for designing protein-like sequences.

### 3.3. The modularity of the weighted CSNs

**Figure 5.**
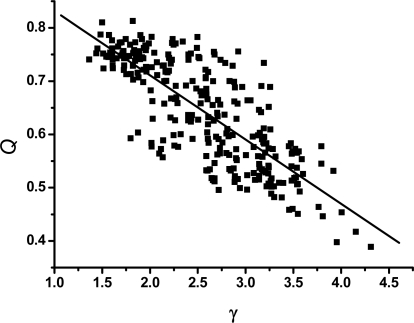
Relationship between the scaling exponent *γ* s of the weighted CSNs and the modularity *Q*s. The data can be fitted by a straight line, with a correlation coefficient of −0.79.

**Figure 6.**
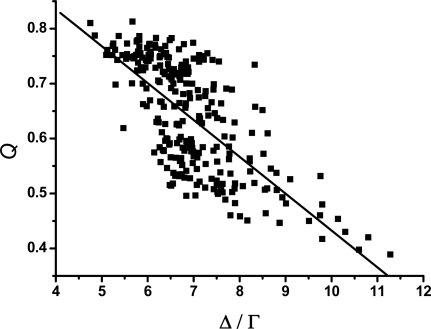
Correlation between the modularity *Q*s and the ratio Δ Γ. The straight line is the fitting line of the data. And the correlation coefficient is −0.70.

Figures 5 and 6 show the relationship between the scaling exponent *γ* s and the modularity *Qs* and the relationship between the ratio Δ/Γ and the modularity *Q*s, respectively. The values of modularity *Q*s are obtained by the MSG-VM algorithm [31,32], and the parameters which are needed in the algorithm can be calculated through the Equations (4) and (5). It can be found from both Figure 5 and Figure 6 that *Q* is more than 0.4 for most weighted CSNs. In addition, as shown in Figure 5, the *γ* s and the *Q*s correlate well with a linear inverse relationship on average. A similar relationship between the ratio Δ/Γ and the modularity *Q*s can be found in Figure 6.

The value of *Q* will be zero for the randomized network [30]. Nonzero values represent deviations from randomness, and in practice it is found that a value of *Q* above 0.3 is a good criterion for a distinct modularity structure [30]. Therefore, the result that most values of *Q*s are more than 0.4 implies that the majority of the weighted CSNs have significant community structures. This indicates the overall topology of the CSN is heterogeneous [69] with subsets of vertices within which vertex-vertex connections are dense, but there are only scant connections between the subsets.

The inverse proportion relationships shown in Figures 5 and 6 may have some interesting implications concerning the modularity mechanism [70] and the topology of the CSNs. In some biological networks, the modularity structure seems to increase the stability, robustness, and flexibility [33,71] of the networks, and recent theory suggests that modularity can be enhanced when the environmental changes over time [72]. From Figure 6, we can find that the larger value of *Q* on average corresponds to the relative smaller value of Δ/Γ. In other words, the sequences that fold relatively fast appear to avoid forming significant community structures in the conformation space generated by the folding process. When addressed in terms of the free energy landscape, the scenario may be more easily understood. In the view of free energy landscape, the behavior of protein folding can be described by a free energy landscape that looks like a funnel [8,9], where there are many different depth minima and various height barriers among them on the coarse surface [8]. In this language, the surface of the energy landscape can be divided into many basin areas, with the deeper minima having large basins of attraction [5,73,74]. Inside a basin and among the basins between which the height of the barriers is small, there are many dynamical accessible routes among the conformations [8]. On contrast, if the barriers between the minima are large, there are a small number of pathways among them. This scene reveals the original source of the modularity structure in the weighted CSNs. Moreover, under the evolution pressure, the sequences of protein are selected to have relatively smooth energy landscape on which there are a small quantity of deep minima and high barriers in order to fold quickly [8,57], in other words, the sequences which fold relatively rapidly avoid to form conspicuous modularity structures in the weighted CSNs.

To obtain the values of the modularity *Q* in the article, we should calculate the weight of the edges in the network, which describes in some sense how the nodes closely connected. We defined the weight of edges according to expression (5). A possible explanation can help us to understand the meaning of the weight of the edges. As pointed out above, the Boltzmann factor of a lattice conformation depicts the relative quantity of the group of the subconformations around the conformation. When two conformations connect in the CSN, it means there are two potential groups connected to each other and the weight of an edge linking two nodes represents the quantity of the possible accessible routes between the corresponding two groups of the subconformations. The larger value of the product of the Boltzmann factor of the two nodes connected by an edge means the more potential accessible routes between them, thus may carefully consider that these two nodes more closely connected.

Finally, several tasks remain to be done in the future. First of all, one may consider the conformation space generated by a three-dimensional (3D) lattice chain and a more reasonable weighting method. In addition, more details about relationship between the topology of the CSN and protein dynamics could be studied to obtain more profound insights into the protein dynamics and the topology of the CSN. Besides, the farther modular analysis of CSN will help us to comprehend expressly the rugged energy landscape. For example, through modular analysis, one may find out more information about the micro-structure on the surface of the energy landscape, such as “local minima” [69]. Finally, one may also consider the rate of transition [75] between the conformations as the weight of edge and the directed network [1] in the modularity analysis in the future.

## 4. Conclusions

Complex network theory was applied to analyze the topological properties of the conformation spaces generated by short two-dimensional HP lattice chains and the underlying free-energy landscape. Scaling behavior is observed in the CSN topology when the weight based on interaction energy of monomers inside the sequences is considered. This result uncovers the importance of the monomer interaction in forming the topology of the CSNs, thus may provide an optional comprehension about the origin of the scale-free property of the CSNs. Moreover, the significant correlation between the scaling exponent *γ* s of the weighted CSNs and the *Z* score, which is often used to measure the thermodynamic stability and kinetic of the sequences, indicates that the global topology of the weighted CSNs has profound connection with the folding dynamic behavior. Finally, the modular structures [22] of the weighted CSNs are also investigated. We find that the sequences that fold relatively faster have a relatively smaller value of the *Q*, which is always applied to quantificationally describe the modular structure of the complex network. A possible physical explain underlying the theory of the energy landscape is also given to discuss this phenomenon.
